# Biocompatibility of Biodentine™ ^®^ with Periodontal Ligament Stem Cells: In Vitro Study

**DOI:** 10.3390/dj8010017

**Published:** 2020-02-08

**Authors:** Duaa Abuarqoub, Nazneen Aslam, Hanan Jafar, Zakariya Abu Harfil, Abdalla Awidi

**Affiliations:** 1 Cell Therapy Center, The University of Jordan, Amman 11180, Jordan; Nazneenaslam66@hotmail.com (N.A.); hanan.jafar@gmail.com (H.J.); 2 Department of Pharmacology and Biomedical Sciences, Faculty of Pharmacy, University of Petra, Amman 11180, Jordan; 3 Faculty of Medicine, The University of Jordan, Amman 11180, Jordan; 4 Faculty of Dentistry, Istanbul Medipol University, Istanbul 34040, Turkey; znabuharfil12@den.just.edu.jo

**Keywords:** Biodentine™, biocompatibility, stem cells, hPDLSCs

## Abstract

Biodentine™ is a tricalcium silicate-based cement material that has a great impact on different biological processes of dental stem cells, compared to other biomaterials. Therefore, we aimed to investigate the optimum biocompatible concentration of Biodentine™ with stem cells derived from periodontal ligament (hPDLSCs) by determining cell proliferation, cytotoxicity, migration, adhesion and mineralization potential. hPDLSCs were treated with Biodentine™ extract at different concentrations; 20, 2, 0.2 and 0.02 mg/mL. Cells cultured without Biodentine™ were used as a blank control. The proliferation potential of hPDLSCs was evaluated by MTT viability analysis for 6 days. Cytotoxicity assay was performed after 3 days by using AnnexinV/7AAD. Migration potential was investigated by wound healing and transwell migration assays at both cellular and molecular levels. The expression levels of chemokines CXCR4, MCP-1 and adhesion molecules FGF-2, FN, VCAM and ICAM-1 were measured by qPCR. The communication potentials of these cells were determined by adhesion assay. In addition, mineralization potential was evaluated by measuring the expression levels of osteogenic markers; ALP, OCN, OPN and Collagen type1 by qPCR. Our results showed significant increase in the proliferation of hPDLSCs at low concentrations of Biodentine™ (2, 0.2 and 0.02 mg/mL) while higher concentration (20 mg/mL) exhibited cytotoxic effect on the cells. Moreover, 2 mg/mL Biodentine™ showed a significant increase in the migration, adhesion and mineralization potentials of the derived cells among all concentrations and when compared to the blank control. Our findings suggest that 2 mg/mL of Biodentine™ is the most biocompatible concentration with hPDLSCs, showing a high stimulatory effect on the biological processes.

## 1. Introduction

Cemented root filling materials provide apical sealing which prevents the transfer of bacterial products and other irritants between root canals and the surrounding periapical tissues. Therefore, these materials must be biocompatible with periradicular tissues [1,2,3].

Calcium hydroxide has been the standard material to maintain pulp vitality ever since 1928, due to the capability to form tertiary dentine. Calcium hydroxide was later replaced by MTA (mineral trioxide aggregate). MTA is a mixture of salts containing dicalcium and tricalcium silicate, calcium sulfate dehydrate, tricalcium aluminate and bismuth oxide [4]. MTA is characterized as a superior root filling material, when compared to other materials, such as Super-EBA, amalgam and intermediate restorative material (IRM), because of its biocompatibility. Since calcium phosphate is precipitated on nucleation sites presented by the hydration products of MTA (particularly the C-S-H gel phase), the process of precipitation is driven by the local increase in concentration of calcium ions and high pH in the vicinity of the MTA [5,6,7]. However, MTA has some drawbacks. It is a time consuming material, since it requires a long time for sealing, ranging from 2 to 4 h [3]. Additionally, it has low compression resistance and flow capacity [7].

Presently, calcium silicate-based materials have become very popular due to their resemblance to mineral trioxide aggregate (MTA). In recent years, the launching of various types of calcium silicate-based materials have increased the demand of these products. One of these has been considered as the core of attention and the main topic of a variety of investigations. This specific replacement material is a calcium silicate-based material named Biodentine™ [8].

Biodentine™ (BD) is a bioactive material that has additional improved characteristics over MTA regarding compatibility, manipulation and hardening [9,10,11]. Its powder components are a mixture of these salts: calcium carbonate, a dicalcium silicate and tricalcium silicate compounds, in addition to oxide filler, iron oxide shade and zirconium oxide. Thus, this mixture makes it a better filling material, when compared to others such as Portland and MTA cement [8]. The hydration kinetics of these components in Biodentine™ has improved its physicochemical properties such as the mechanical strength, speed of curing, the initial and the final setting time [8,12,13]. Biodentine™ has been considered as a promising pulp capping material that can be used in the field of restorative dentistry to replace the dentine. Additionally, Biodentine™ has a great potential to be used in the field of endodontic surgery where it can be used as retrograde filling material. Moreover, it has multi-uses in the endodontic repair field such as apexification, resorptive lesions and root perforations [1,2,3].

Biodentine™ is a member of the larger biocermaic group, in which this latter group has great potential towards the differentiation and proliferation of dental stem cells. These cells are known for their clonogenic capability and potential to differentiate into multilineage [3]. Apart from dental pulp stem cells (DPSCs), different types of dental stem cells are under investigation for their multipotent effects; stem cells from human exfoliated deciduous teeth (SHED) [14], periodontal ligament stem cells (PDLSCs) [15], dental follicle progenitor cells (DFPCs) [16], alveolar bone-derived MSCs (ABMSCs) [17], stem cells from apical papilla (SCAP) [18], tooth germ progenitor cells (TGPCs) [19] and gingival MSCs (GMSCs) [20].

Periodontal ligament stem cells (PDLSCs) reside in the perivascular space around teeth and possess multipotent differentiation ability such as osteogenesis, adipogenesis and chondrogenesis. These derived cells also have a higher proliferation rate when compared to skeletal stem cells derived from bone marrow which makes them a promising source for periodontal ligament regeneration. PDLSCs are able to suppress immune responses and inflammatory reactions meaning that they will not provoke an immunogenic reaction in case of being of an allogenic origin [1]. PDLSCs are considered a highly promising stem cell population for repair and regeneration of periodontium and other tissues [15].

Optimum concentrations of Biodentine™ for regeneration of pulp–dentine complex for clinical application have been reported [21,22,23,24,25,26,27,28]. DPSCs extracted from permanent teeth have been evaluated for regenerative potential in conjunction with Biodentine™ [29]. In addition, peridontal ligament(PDL) fibroblasts showed an increased ability of cell adhesion through the expression of focal contacts after treatment with Biodentine™. However, there is no previous investigation that has evaluated the optimum concentration of Biodentine™ for the regeneration of hPDLSCs. Our study aims to investigate the stimulatory or cytotoxic effect of Biodentine™ on hPDLSCs, in vitro. We will particularly determine the optimum concentrations of Biodentine™ on proliferation, cytotoxicity, migration, adhesion and the mineralization potential of hPDLSCs.

## 2. Materials and Methods

### 2.1. Preparation of Biodentine™

Biodentine™ was prepared as previously described [6]). Briefly, Biodentine™ capsule was mingled with its own solution provided by the manufacturer. Following that, a step of dryness and sterilization of the mix was performed by using an oven at 60 °C for 15 min. Then, the pestle and mortar were used to grind the dried powder. To prepare the concentration, 20 mg/mL of Biodentine; the prepared dried powder was added to 50 mL of the cell culture alpha MEM essential medium (α-MEM, Gibco). The medium was vortexed until it was completely suspended. Following that, the medium was filtered twice by using 70 µM cell strainer (BD, Franklin Lakes, NJ, USA). A serial dilution of 10 folds was performed to prepare different concentrations of Biodentine™; 2, 0.2 and 0.02 mg/mL. Cell culture medium without Biodentine™ was used as a blank control.

### 2.2. Sample Collection

Human third molar samples were collected from healthy donors (18, 19 and 21 years), according to the Institutional Review Board (IRB) guidelines from the Cell Therapy Center/University of Jordan (IRB/06/2018) and approved on 13 March 2018. All human participants signed informed consent before their tooth donation.

### 2.3. Cell Culture

Cells were isolated by using enzymatic digestion method, as described previously [30]. Briefly, teeth were disinfected with PBS (1X) thrice followed by incubation for five minutes. PDL tissue was scraped from mid third level of root of each third molar, then tissue was incubated in a solution containing two digestive enzymes; 4 mg /mL dispase (GIBCO, Waltham, MA, USA) and 3 mg/mL collagenase type I (GIBCO, USA) for 1 h at 37 °C. Following that, single-cell suspensions were filtered through a 70 μm strainer (BD Biosciences, Franklin Lakes, NJ, USA). Cells were centrifuged at 200× *g* for 5 min. Pellet was re-suspended in 1 mL of culture media and cells were seeded in 6-well plates using alpha-modification of Eagle’s Medium [(a-MEM, GIBCO, USA), supplemented with 2 mM L-glutamine (Invitrogen, Carlsbad, CA, USA), 100 mg/mL streptomycin (Invitrogen, Carlsbad, CA, USA), 100 units/mL penicillin (Invitrogen, Carlsbad, CA, USA) and 0.25 mg/mL Amphotericin B (Invitrogen Carlsbad, CA, USA)]. For monolayer generation of primary culture, 5% concentration of platelet lysate (PL) was used while serum free media was used as a negative control. The cells were incubated at 37 °C in 5% CO_2_ incubator and culture medium was changed every three days until the cells reached 70–80% confluency.

The derived human periodontal ligament stem cells (hPDLSCs) were isolated and characterized according to expression of MSC profile and differentiation potential [30].

### 2.4. Cell Proliferation Assay (MTT)

To explore the impact of using Biodentine™ on the proliferation rate of hPDLSCs, MTT assay was performed. A total of 2500 PDL cells at passage number 3 were seeded onto a 96-well plate in different concentrations of Biodentine™ and a blank control. Culture medium was changed every 3 days and the cells were incubated for 6 days at 37 °C 5% CO_2_. CellTiter 96^®^ Non-Radioactive Cell Proliferation Assay (MTT) (Promega, Madison, WI, USA) kit was used to measure the optical density at 570 nm using multiplate reader (Glomax, Promega, Madison, WI, USA).

### 2.5. Cytotoxicity of Biodentine™

To determine whether Biodentine™ has a cytotoxic effect on hPDLSCs, cells were stained by Annexin V/7AAD dye.

Human periodontal ligament stem cells (hPDLSCs) at passage 3, (1 × 10^5^ cells/well) were cultured in 6-well plates and treated with different concentrations of Biodentine™ along with a blank control. The experiment was conducted for 3 days. Next, cells were trypsinized and incubated with Annexin V/7AAD dyes (BD, Biosciences) in concentrations according to manufacturer’s suggestion (BD, Franklin Lakes, NJ, USA). Stained cells were resuspended with PBS, after a centrifugation step at speed of 300× *g* for 5 min. The expression profile was analyzed by FACS Diva 8 (FACS Canto BD, Franklin Lakes, NJ, USA).

### 2.6. Migration Potential

To evaluate the influence of using Biodentine™ on the migration potential of the derived hPDLSCs, wound healing (Scratch) and transwell migration assays were performed.

### 2.7. Wound Healing Assay (Scratch Assay)

As previously described [31], 1 × 10^5^ cells/well were cultured in 6 well plates, with culture medium and allowed to reach complete confluent monolayer. Before wound infliction, starvation media without serum were added to hPDLSCs and incubated for 24 h. Then, a wound (scratch) was inflicted by using 200 µL pipette tip, through 100% confluent cells followed by a washing step with phosphate buffered saline (PBS) to remove cell debris. Different concentrations of Biodentine™ were added along with a blank control and experiment was conducted for 24 h (1 day). The progress of wound closure was observed by using phase-contrast microscope (Zeiss, Oberkochen, Germany) and photographs of the scratch were taken at two different time points; before scratching 0 h and after 24 h of wound infliction.

### 2.8. Transwell Migration Assay

Transwell system (Corning, New York, NY, USA) composed of two chambers; 8 mm pore size and 6.5 mm diameter, were used to evaluate the migration potential of the derived cells. Briefly, 5 × 10^4^ hPDLSCs at passage 3 were seeded in the top chamber in 200 µL of serum free alpha-MEM. Then, 700 µL of cell culture media containing different concentrations of Biodentine™ and a blank control were added to the lower chambers. Cells were incubated for 24 h at 37 °C in 5% CO_2_ incubator. Non-migrating cells were taken off from the top surface of the filter by using a cotton swab, while the migrated cells, traversed to the bottom chamber membrane, were fixed with 4% paraformaldehyde for 10 min. After fixation, membranes were cut by using a blade and stained with DAPI (Life technologies, Carlsbad, CA, USA). The number of migrated cells was counted in five random fields per filter observed at 200× magnification under fluorescent microscope (Zeiss, Oberkochen, Germany).

### 2.9. Adhesion Assay

To study to the influence of Biodentine™ on the adhesive potential of the treated cells, adhesion assay was performed.

Type 1 collagen (40 mg/L in PBS) was used to coat 96-well plates overnight at 4 °C. Different concentrations of Biodentine™ and a blank control were mixed with the cells prior to plating. Cell density was kept at 1 × 10^5^ cells/mL and 100 µL of the cell suspension were added into each well, incubated for 1 hr at 37 °C in 5% CO_2_, followed by a fixation step with 4% formaldehyde for 20 min. Then, the fixed cells were stained with 0.1% crystal violet for 30 min. After that, 10% acetic acid was added to the wells as a solubilization step. Optical density was measured at 595 nm on a multiplate reader (Glomax, Promega, Madison, WI, USA).

### 2.10. Migration Potential at Molecular Level

To determine the effect of Biodentine™ on the expression profile of migratory genes, the expression levels of chemokines (MCP-1, CXCR4) and adhesion molecules (ICAM-1, FN, VCAM-1 and FGF2) were determined by using qPCR.

hPDLSCs were cultured with different concentrations of Biodentine™ and a blank control for 3 days (72 h). Total RNA was extracted from cells using Trizol reagent (Life Technologies, USA) and then was treated with DNase I (RNase-free, RQ1; Promega, Madison, WI). One microgram of total RNA was used to synthesize cDNA by using Vilo superscript (Life technologies, USA). Q-PCR analyses were performed with SYBR Green PCR master mix reagent (Promega, USA) using CFX96 (Bio rad, Hercules, CA, USA). The PCR conditions were; denaturing 95 °C for 10 s, annealing 60 °C for 15 s and extension 72 °C for 10 s, and repeated in a 35-PCR cycle. The normalization was done to the expression level of a house keeping gene GAPDH, in addition to the cells harvested at day zero of the treatment. Specific primer sets for analysis are listed in Table 1.

### 2.11. Mineralization Potential (Osteogenic Differentiation)

To evaluate the effect of Biodentine™ on the osteogenic differentiation potential, the expression levels of osteogenic markers (OCN, OPN, ALP, Cbfa-1 and Collagen type), were measured by using qPCR.

hPDLSCs at passage 3 were cultured in 6-well plate (1 × 10^5^ cells/well) with cell culture medium and allowed to become 60–80% confluent. After that, culture medium containing different concentrations of Biodentine™ was added and cell culture medium was kept as a negative control. Cells treated with osteogenic differentiation media (Invitrogen, Carlsbad, CA, USA) were used as a positive control. All cells were incubated with their respective culture media for 21 days.

Total RNA was extracted from cells using Trizol reagent (Life Technologies, USA). One µg of total RNA was used to synthesize cDNA by using Vilo superscript (Life technologies, USA). Q-PCR analyses were performed with SYBR Green PCR master mix reagent (Bio-rad, Hercules, CA, USA) using CFX96 (Bio-Rad, USA). The PCR conditions were: denaturation 95 °C for 10 s, annealing 58 °C for 15 s, and extension 72 °C for 10 s of each PCR cycle and repeated for 35 cycle. The relative amount or fold change of the target gene was normalized relative to the level of human housekeeping gene cyclophilin gene (PPIA) and the day zero of the control untreated cells. The following specific primers sets for analysis are listed in Table 2.

### 2.12. Statistical Analysis

GraphPad Prism and Microsoft windows Excel were used for data analysis. All experiments were run in triplicate in three independent experiments (n = 3). And results were expressed as means ± standard deviations (SD). A paired t-test and one-way ANOVA analysis were used to determine the statistical differences among all assays. (Significance assumed for *p* < 0.05).

## 3. Results

### 3.1. Proliferation Assay (MTT)

**Figure 1:**
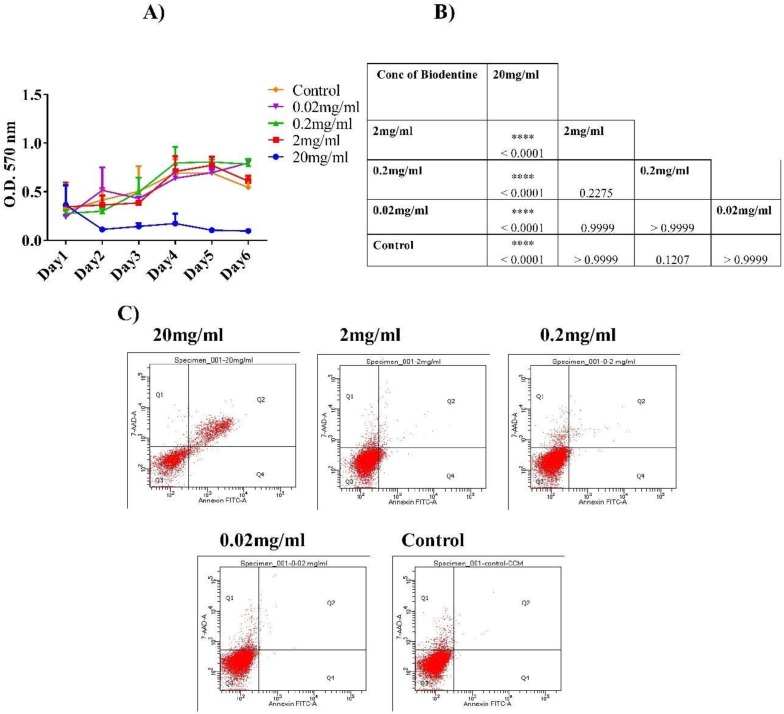
Cell proliferation and cytotoxicity of human periodontal ligament stem cells hPDLSCS treated with Biodentine™. (**A**) Cell proliferation (MTT) assay of hPDLSCs treated with different concentrations of Biodentine™ (20, 2, 0.2, 0.02 mg/mL) and blank control. (**B**) A representative table showing the statistical differences among all concentrations for 6 days of MTT assay Biodentine™ (*p* < 0.05). (**C**) Flow cytometric analysis of hPDLSCs stained with Annexin V/7AAD stain. The four quadrants of dot plot indicate the percentage of: necrotic cells (Q1), late apoptotic cells (Q2), viable cells (Q3) and early apoptotic cells (Q4). (**** *p* < 0.00005, *** *p* < 0.0005, ** *p* < 0.005, * *p* < 0.05).

A significant increase in the proliferation rate of hPDLSCs was observed when cells were treated with the lower concentrations of Biodentine™ (2, 0.2 and 0.02 mg/mL) (*p* < 0.05). However, 20 mg/mL showed a significant decrease in hPDLSCs proliferation, suggesting cytotoxic effect of Biodentine™ at higher concentration (*p* < 0.05) (Figure 1A,B).

However, insignificant difference was observed among different concentrations of Biodentine™ (2, 0.2, 0.02 mg/mL) in comparison to their control and untreated cells.

### 3.2. Cytotoxic Assay

Apoptosis analysis was performed using Annexin V/7AAD dyes. The results showed that at higher concentration (20 mg/mL), Biodentine™ exerted anti-proliferative effect on cells in a significant manner (*p* < 0.05). However, hPDLSCs cultured in the 2, 0.2 and 0.02 mg/mL of Biodentine™ showed high proliferation rates with low percentages of necrotic and apoptotic cells indicating a minimal cytotoxic effect, without any statistical differences (Figure 1C).

At this stage, we measured the pH value of 20 mg/mL of Biodentine™ which was around 11.58. Perhaps this highly alkaline pH caused detrimental effect on the viability of hPDLSCs. Due to the high cytotoxic effect of 20 mg/mL of Biodentine™, it was excluded from this study at this point.

### 3.3. Migration Potential Assays

#### 3.3.1. Wound Healing (Scratch Assay)

**Figure 2:**
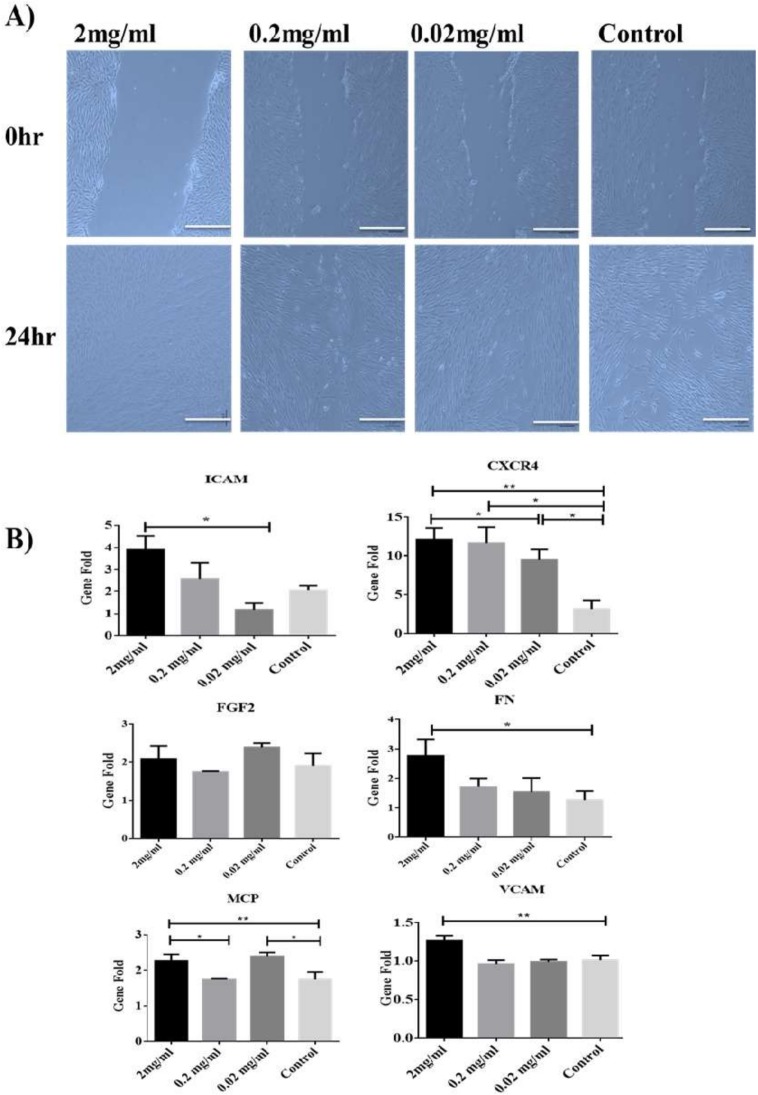
Effect of Biodentine on wound healing. (**A**) Microscopic photographs (100×) of PDLSCs treated with different concentrations of Biodentine™ (2, 0.2, 0.02 mg/mL) and controls. (**B**) Measurement of the expression levels of migration genes CCXR4, MCP, FN, FGF2, VCAM and ICAM-1, among all treated groups after 24 h of wound closure by using qPCR. (** *p* < 0.005, * *p* < 0.05).

To find out the role of Biodentine™ on the migration potential of the derived cells, wound healing (Scratch) assay was performed. It was evident that hPDLSCs treated with low concentrations of Biodentine™ were able to narrow the distance of wound after 24 h. However, there were some minor variations in the closure of the wound among different concentrations of Biodentine™ and the blank control (Figure 2A).

At molecular level, our data showed that 2 mg/mL was able to induce the expression of chemokines (MCP-1 and CXCR4) and adhesion genes (VCAM-1, ICAM-1 and FN) in a significant manner (*p* < 0.05) among all treatments compared to the control group. However, for FGF2 (adhesion molecule), all treated and untreated groups showed the similar expression pattern of the latter molecule (Figure 2B).

#### 3.3.2. Transwell Membrane Assay

**Figure 3:**
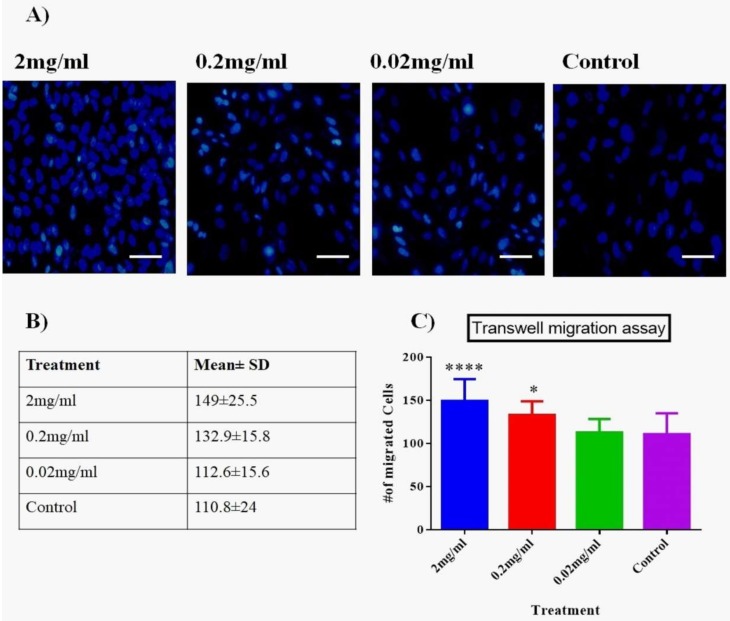
Two-chamber Transwell system. (**A**) Evaluation of migration potential using two-chamber Transwell system of hPDLSCs treated with media containing different concentrations of Biodentine™ and controls (magnification 200×). (**B**) A representative table of the number of migrating cells among all treated group. Data are shown as mean ± SD. (**C**) A statistical analysis of the migrating cells among all treated groups (*p* < 0.05). (**** *p* < 0.00005, * *p* < 0.05).

To determine the effect of Biodentine™ on vertical migration potential within the tissues, Transwell system (two-chambers) was used. Interestingly, our results showed that both 2 and 0.2 mg/mL of Biodentine™ showed a significant difference in the number of migrating cells compared to the control (*p* < 0.05) and 2mg/mL has significantly stimulated the migration ability of cells (*p* < 0.05) (Figure 3).

### 3.4. Adhesion Assay

**Figure 4:**
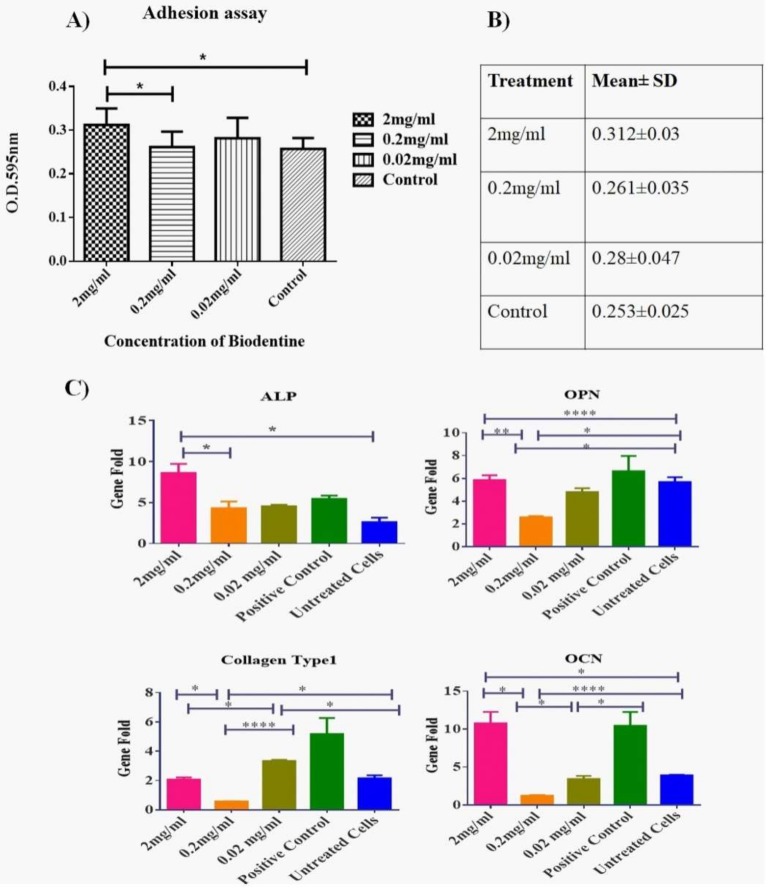
Effect of Biodentine™ on the adhesion and mineralization potential of hPDLSCs treated with Biodentine™. (**A**) A statistical analysis of the number of the adherent hPDLSCs treated with different concentrations of Biodentine™ (2, 0.2, 0.02 mg/mL) and blank control. A significant difference in the number of adherent cells was observed among the different treated groups P (<0.05). (**B**) A representative table of the mean ± SD of adherent cells among all treated groups (**C**) The expression levels of osteogenic markers; ALP, OCN, OPN and Collagen type 1 of Biodentine™ treated and control groups. (**** *p* < 0.00005, *** *p* < 0.0005, ** *p* < 0.005, * *p* < 0.05).

Biodentine™ effect on the adhesive ability of hPDLSCs was measured. As shown in Figure 4A,B, 2 mg/mL showed a significant rise in the number of adhesive cells as compared to all other concentrations and the blank control (*p* < 0.05).

### 3.5. Mineralization Potential

The impact of Biodentine™ on the differentiation capability of hPDLSCs was measured by detecting the expression of osteogenic markers after culturing cells with Biodentine™ for 21 days.

Our data showed that the expression levels of osteogenic genes ALP, OCN and OPN were up regulated significantly in a similar manner as the positive control (cells treated with osteogenic differentiation media) (*p* < 0.05). Two mg /mL showed the highest expression profile among all other treatments, expect for Collagen type 1 gene where the treated cells did not record any significant variations on the expression level of the latter gene in comparison to the untreated cells (Figure 4C).

## 4. Discussion

The correlation between stem cells and the filling materials is very important in terms of tissue regeneration and repair after endodontic surgery. Therefore, the ideal root filling material must be biocompatible with low cytotoxicity. In clinical conditions, the biocompatibility of a root filling material must be acceptable, in order to minimize the negative effect of the filling biomaterial on the surrounding periodontal tissues [32].

Biodentine™ (BD) is relatively a new calcium silicate-based cement, synthesized to be used in direct contact with the periodontal ligament, which is crucial in conservative therapy. It has been suggested that the attachment of cells to the root surface is a critical and complicated dynamic process, which has implications in biological processes, such as cell proliferation and differentiation [33]. The effect of Biodentine™ on the adhesive ability of fibroblasts of PDL has been reported (40). However, information regarding the optimum concentration of Biodentine™ for stimulation or inhibition of the biological processes of hPDLSCs is lacking. Therefore, we aimed to explore the effect of Biodentine™ on hPDLSCs derived from the surrounding periodontal tissue [34]. Thus, the cytotoxic and proliferative function of different concentrations of Biodentine™ was examined on hPDLSCs. Cell viability and cytotoxicity are two key factors that are important to determine the biocompatibility of Biodentine™. Interestingly, our MTT and cytotoxicity results showed that Biodentine™ enhanced the proliferative activity and decreased the cytotoxicity of the derived cells at low concentrations 2, 0.2 and 0.02 mg/mL in a similar manner. While high concentration 20 mg/mL resulted in a reduction of cell growth leading to an increase in cell death percentages, compared to the low concentrations. These data are consistent with previously published data [31,32,35].

It has been reported that higher pH values of Biodentine™ lead to an increased influx of iron traces and calcium ions in the extracellular environment [36]. Elevated extracellular calcium levels have been associated with promotion of proliferation, migration and mineralization potential of MSCs [37]. On the other hand, higher or imbalanced iron levels can be toxic for the viability of cells [38]. Perhaps the high iron traces are the reason for cytotoxic effect of higher concentration of Biodentine™ in our study.

The wound healing process is divided into three stages, starting with the inflammatory and fibrogenic stage, followed by the regeneration phase and the last one is the remodeling stage. Through all these stages, many biological processes are required for tissue growth and repair such as proliferation, migration and adhesion, so that new progenitor cells can start to regenerate the odontoblast-like cells in order to compensate the lost cells at the border line between pulp and dentin [34].

Additionally, both the cellular processes (migration and adhesion) play a key role in the maintenance of tissue homeostasis, repair and regeneration. Therefore, to make these goals achievable, cells must be able to migrate and adhere to the targeted sites for regeneration and development of functional tissues [33,39]. As previously described [40], dental stem cells respond to these injuries by these cellular processes; proliferation, differentiation, migration and adhesion in order to repair and replace the damaged tissues.

Remarkably, in our study, Biodentine™ has an overall stimulatory effect on the derived cells through the significant enhancement of their migration, adhesion and mineralization potential. Additionally, these processes were stimulated by up-regulation of the expression of certain chemokines and adhesion molecules, whereas the mineralization potential was activated through the up-regulation of certain osteogenic markers.

From the migration assays (wound healing and transwell assay), it was evident that hPDLSCs treated with low concentration of Biodentine™ had significant migration capability at cellular level, compared to the untreated control cells. Interestingly, our results are similar to the previous published studies in respect of treating stem cells with Biodentine™ [32,41] and opposite to the use of other biomaterials such MTA and resin, where both had a negative effect on producing dentine bridge synthesis [42,43].

On the other hand, the impact of Biodentine™ was also examined at gene level for the markers involved in the wound healing and migration potential of the derived cells such as: CXCR4, MCP-1, FN, FGF2, VCAM and ICAM-1.

CXCR4 increases the migration potential towards endothelial cells in skin wounds, by accelerating the healing process in a short period of time. Additionally, FGF2 is involved in the differentiation process, and the regulation of chemokine expression, promotes the tissue regeneration. Whereas, MCP-1, a chemokine regulator, enhances the recruitment of adventitial vascular progenitor cells to the injured lesions [44,45].

Our results showed that 2 mg/mL Biodentine™ was able to promote the expression of these chemokines CXCR4 and MCP-1 and adhesion molecules FN, ICAM-1 and VCAM-1, significantly when compared to the untreated control cells. And no changes were observed in the expression of the FGF2 marker. Importantly, these results are in agreement with previously published data [41].

Regarding the osteogenic potential, cells treated with 2 mg/mL Biodentine™ were able to promote the expression of osteogenic markers such as: ALP, OPN and OCN in a significant manner, except for the Collagen type 1. These markers were up-regulated in a similar manner to the cells induced with osteogenic media, indicating a high mineralization potential. These data are also in agreement with previously published data [32,35].

## 5. Conclusions

We conclude that Biodentine™, at concentration of 2 mg/mL is a biocompatible material and can be used as root filling material, due to its role in increasing the proliferation, migration, adhesion and differentiation potential of the derived hPDLSCs at both cellular and molecular levels. These properties make it a very promising material to be used in tissue repair and regeneration.

## Figures and Tables

**Table 1:** Primer sequence of chemokines and adhesion molecules (migration genes).

Gene	Forward	Reverse
CXCR4	TACACCGAGGAAATGGGCTCA	AGATGATGGAGTAGATGGTGGG
FGF2	CGTGCTATGAAGGAAGATGGA	TGCCCAGTTCGTTTCAGT
MCP-1	CCAAAGAAGCTGTGATCTTCAA	TGGAATC CTGAACCCACTTC
FN	TCCTTGCTGGTATCATGGCAG	AGACCCAGGCTTCTCATACTTGA
ICAM-1	ATCCATCCCACAGAAGCCTTCCTGC	GCCCACCTCCAGGAGGTCAGGGGTGT
VCAM	CATGACCTGTTCCAGCGAGG	CATTCACGAGGCCACCACTC
GAPDH	CCTGCACCACCAACTGCTTA	GGCCATCCACAGTCTTCTGAG

**Table 2:** Primer sequence of osteogenic markers.

Gene	Forward	Reverse
ALP	AGTAGGGCCTGGATC TTC TT	CTGCTTCTCAGTCAG AAGGT
OPN	TGCAGCCTTCTCAGCCAA	GGAGGCAAAAGCAAATCACTG
OCN	GACGAGTTGGCTGACCACA	CAAGGGGAAGAGGAAAGAAGG
Collagen type 1	GGAGATGATGGGGAAGCTGG	TTGGCACCATCCAAACCACT
PPIA cyclophillin	TCCTGGCATCTTGTCCATG	CCATCCAACCACTCAGTCTTG
